# Oral Rabies Vaccination of Small Indian Mongooses (*Urva auropunctata*) with ONRAB via Ultralite Baits

**DOI:** 10.3390/v13050734

**Published:** 2021-04-23

**Authors:** Are R. Berentsen, Israel L. Leinbach, Mel J. Rivera-Rodriguez, Amy T. Gilbert

**Affiliations:** 1 United States Department of Agriculture, Animal and Plant Health Inspection Service, Wildlife Services, National Wildlife Research Center, 4101 LaPorte Ave., Fort Collins, CO 80521, USA; amy.t.gilbert@usda.gov; 2 United States Department of Agriculture, Animal and Plant Health Inspection Service, Wildlife Services, National Wildlife Research Center, 210 Amauulu Rd., Hilo, HI 96720, USA; Israel.L.Leinbach@usda.gov; 3 United States Department of Agriculture, Animal and Plant Health Inspection Service, Wildlife Services, 602 Duncan Dr., Auburn, AL 36849, USA; Mel.j.rivera.rodriguez@usda.gov

**Keywords:** *Urva auropunctata*, ONRAB, rabies, small Indian mongoose, Ultralite bait

## Abstract

The Ontario Rabies Vaccine (ONRAB) is a human adenovirus rabies glycoprotein recombinant oral vaccine immunogenic for small Indian mongooses when delivered by direct instillation into the oral cavity. We offered Ultralite baits containing ~1.8 mL 10^9.5^ TCID_50_ ONRAB oral rabies vaccine to 18 mongooses, while 6 mongooses were offered identical baits in placebo form. We collected sera from individual mongooses at days 0, 14 and 30 post vaccination (pv) and quantified rabies virus neutralizing antibodies (RVNA) using the rapid fluorescent focus inhibition test, with titers greater than or equal to 0.1 IU/mL considered positive. All study subjects were RVNA negative prior to bait offering. Bait consumption was variable: all 6 sham and 13 of 18 (72%) treatment animals consumed/punctured the baits offered. By day 30 pv, RVNA were detected among 11 of 13 (84.6%) of treatment mongooses that consumed/punctured baits, whereas sham-vaccinated mongooses remained RVNA negative throughout the study. We conclude ONRAB is immunogenic for mongooses by Ultralite bait delivery, although the bait design may need further optimization.

## 1. Introduction

The small Indian mongoose (*Urva auropunctata*; formerly *Herpestes auropunctatus*) is a rabies reservoir on Puerto Rico and other Caribbean Islands [1]. In the United States and Europe, oral rabies vaccination (ORV) is an important tool in managing rabies in wild carnivores [2,3]. However, no oral rabies vaccine is licensed for use with mongooses. The product currently licensed for use with raccoons (*Procyon lotor*) and coyotes (*Canis latrans*) in the United States (RABORAL V-RG, Boehringer Ingelheim Animal Health, Athens, GA, USA) is reportedly not immunogenic for mongooses [4]. However, a vaccine in Europe (RABITEC; Ceva Santé Animale, Dessau Rosslau, Germany) registered for red foxes (*Vulpes vulpes*) and raccoon dogs (*Nyctereutes procyonoides*) has been reported as immunogenic for mongooses [5]. The Ontario Rabies Vaccine (ONRAB; Artemis Technologies, Inc., Guelph, ON, Canada) is a human adenovirus rabies glycoprotein recombinant oral vaccine licensed for use with striped skunks (*Mephitis mephitis*) in Canada, and has been under experimental use in the United States since 2011 [6,7,8,9]. ONRAB was immunogenic for small Indian mongooses when delivered by direct instillation into the oral cavity [10], but a bait format for oral delivery has not been attempted. Our objective was to conduct a preliminary evaluation regarding the immunogenicity of ONRAB delivered to mongooses via Ultralite baits used to target other wild carnivore reservoirs and vectors of rabies virus in North America.

## 2. Materials and Methods

### 2.1. Study Area

We conducted this study at the United States Department of Agriculture, Animal and Plant Health Inspection Service, Wildlife Services, National Wildlife Research Center Hawaii field station, Hilo, Hawaii, USA. Hawaii is considered free of animal rabies.

### 2.2. Animal Capture and Husbandry

We live captured mongooses in cage traps (Tomahawk Trap Co., Hazelhurst, WI, USA) and transported them to the field station where they were housed individually in 60 × 60 × 40 cm^3^ stainless steel cages. Mongooses were held in acclimation for five to seven days prior to study initiation. Mongooses were maintained on a daily ration of ~50 g commercial dry cat food, supplemented twice weekly with commercial raw chicken. Water was available ad libitum. Ambient temperature in the laboratory was maintained at 24–26 °C with a 12 h/12 h day/night cycle to mimic mongoose diurnal activity periods. Humidity in the laboratory ranged from 60 to 90%. Individual mongoose identification was maintained throughout the study by cage labels containing the cage number and individual animal microchip number. This study was conducted under Animal Biosafety Level 2 conditions.

### 2.3. Bait Description

**Figure 1:**
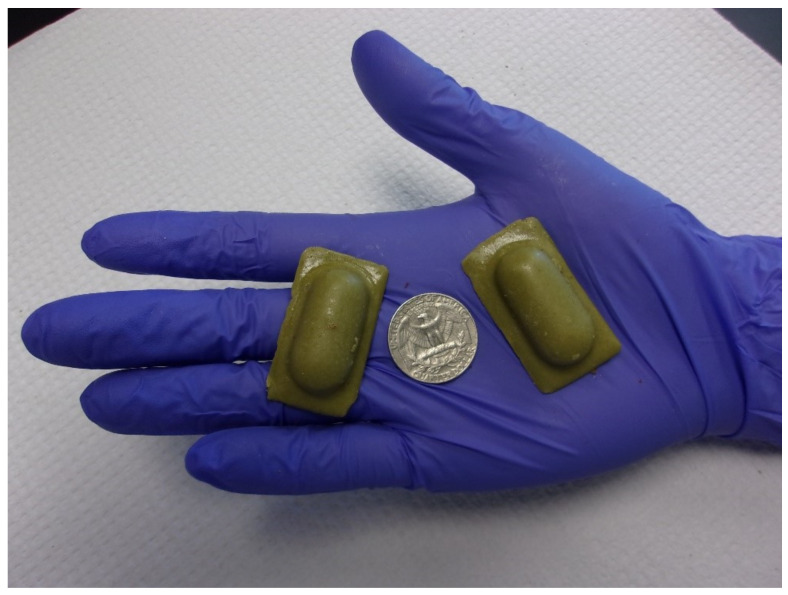
Ultralite ORV baits coated with external bait matrix.

The Ultralite bait is composed of a 30 × 14 × 10 mm^3^ elongated oval foil blister pack with a rectangular lip extending to 40 × 20 mm^2^ ([6], Figure 1). Previous research found cheese-flavored Ultralite baits were preferred by mongooses in field trials in comparison to coconut or fish-flavored baits [11]. Each cheese-flavored bait contained ~1.8 mL 10^9.5^/mL TCID_50_ of ONRAB (Bait lot number OTF 18–26, Manufactured 26 March 2018, Artemis Technologies, Inc., Guelph, ON, Canada). Cheese-flavored control baits contained an equivalent volume of sterile water.

### 2.4. Bait Offering

We offered each of the 18 mongooses (9M, 9F) a single ONRAB bait and each of the six other mongooses (3M, 3F) a single placebo bait for 24 h along with their daily food ration in a free-choice test. Animals were not fasted prior to bait offering. Baits not punctured or chewed after 24 h were considered failed delivery. All baits were removed after 24 h. During the 24 h of bait offering, we made up to three attempts to replace baits recovered immediately under the animal’s cage.

### 2.5. Sample Collection

We anesthetized mongooses prior to vaccination and on days 14 and 30 post vaccination (pv) via inhalation of isoflurane gas or intramuscular injection of 5 mg/kg Telazol^®^ (tiletamine/zolazepam; Zoetis, Inc., Kalamazoo, MI, USA) and collected blood samples as previously described [10]. Serum was separated from whole blood and decanted into cryovials and stored at −80 °C until analysis.

### 2.6. Sample Analysis

We shipped sera to the Rabies Laboratory at Kansas State University (Manhattan, KS, USA) where rabies virus neutralizing antibodies (RVNA) were quantified using the rapid fluorescent focus inhibition test [12]. Titers reported as greater than or equal to 0.1 IU/mL were considered RVNA positive by comparison to a standard rabies immune globulin (SRIG; WHO lot 2) positive control.

### 2.7. Statistical Analysis

Geometric mean (SE) titer values were calculated using Microsoft Excel. Supplementary figures were generated using the program R [13]. Treatment animals with RVNA titers < 0.1 IU/mL on days 14 and 30 pv were assigned a value of 0.05 IU/mL for the purposes of geometric mean calculations.

## 3. Results

All study subjects were RVNA negative prior to bait offering. All (6/6) sham animals and 13/18 (72%; 95% CI 49–88%; 7M, 6F) treatment animals punctured or chewed the bait within the 24 h offering period. By day 14 pv 7/18 (39%; 95% CI 20–61%) of treatment, animals (4M, 3F) were RVNA positive. By day 30 pv, 11/18 (61%; 95% CI 39–80%) of treatment animals (6M, 5F) were RVNA positive. Two treatment animals (1M, 1F; 15%; 95% CI 3–33%) that chewed/punctured baits remained RVNA negative on days 14 and 30 pv. All sham animals and five treatment animals that did not puncture/consume baits remained RVNA negative during the study. Geometric mean (SE) RVNA titers among treatment animals were 0.1 (1.1) and 0.4 (1.3) IU/mL for days 14 and 30 pv, respectively (Table 1). Individual RVNA serology results are presented in Table 2.

## 4. Discussion

Our research suggests that ONRAB is immunogenic for mongooses when delivered via the Ultralite bait and 84.6% of treatment animals that consumed/punctured baits demonstrated induction of RVNA by day 30 pv. However, overall bait consumption suggests further research into bait optimization may be required prior to field evaluation. For example, of the 18 treatment mongooses in this study, 2 mongooses that punctured/chewed baits did not demonstrate RVNA seroconversion, suggesting inefficient oral contact with the vaccine or vaccine spillage from the bait during handling, and 5 treatment animals did not interact with baits at all. It is notable that day 14 and day 30 pv RVNA titers were reduced following bait presentation versus DIOC application of ONRAB to individual mongooses across independent studies with identical vaccine dose and serological methods (Figure S1 [10]). The fact that overall RVNA titers were lower following vaccine delivery by bait when contrasted with DIOC delivery was expected [14] lends further evidence to support the notion that while ONRAB may be immunogenic for mongooses, the bait used to deliver the vaccine needs refinement. Additional refinement of the bait attractant(s) is important based on the results of this study, as 20% of the mongooses did not interact with the baits in this free-choice setting. Modifying the bait structure and shape (e.g., soft and more cylindrical) to suit the narrow shape of the mongoose mouth could facilitate bait handling during consumption to reduce potential for vaccine spillage and/or inefficient oral contact. This study was conducted as a free-choice test with well-fed study subjects, and bait uptake behavior by free-ranging mongooses in the field may be different based on the resources available.

In considering the threshold of 0.5 IU/mL established to assess rabies vaccination in wildlife [15], 3/18 (17%) of mongooses on day 14 pv, and 7/18 (39%) on day 30 pv demonstrated RVNA equal or greater than 0.5 IU/mL. The range of RVNA values found in this study (0.1–10 IU/mL, 30 days pv) raises questions of whether mongooses on the lower end of the range (i.e., <0.5 IU/mL) would survive post vaccination virus challenge. Previous research using an experimental vaccine (SPBNGA-S; [4]) suggests that mongooses on the higher end of this range (≥0.5 IU/mL) may survive a lethal rabies virus challenge but at the lower end, the degree of protection remains uncertain without further efficacy evaluation. At this stage in research and development, bait refinement would likely precede a captive or field efficacy evaluation.

Additional areas of future research may include immunopathology of the tonsils and oral cavity to evaluate variable vaccine uptake by mongooses in the laboratory [16] as well as behavioral studies documenting mongoose–bait interaction. Lastly, biomarkers could be used to provide sensitive and quantitative estimates of bait uptake to aid in optimization for this species [17]. Limited field trials with a placebo ORV bait targeting mongooses have been conducted [11,18], but fewer data are available regarding uptake of ONRAB Ultralite baits by free-ranging mongooses. The control of rabies virus circulation in mongooses in areas like the Caribbean is an exciting new frontier for adaptation of ORV strategies, methods and products for this invasive tropical reservoir host.

## Figures and Tables

**Table 1:** Geometric mean (SE) RVNA titer (IU/mL).

Group	Day 0	N	Day 14	N	Day 30	N
Treatment	<0.1	18	0.1 (1.1)	7	0.4 (1.3)	11
Sham	<0.1	6	<0.1	6	<0.1	6

**Table 2:** Individual RVNA titers in 24 small Indian mongooses (*Urva auropunctata*) prior to treatment and days 14 and 30 post-offering of Ontario Rabies Vaccine Ultralite baits. * Sample collected on day 13.

Animal ID	Group	Sex	RVNA Titers (IU/mL)			Bait Consumed/Punctured after 24 h? (Y/N)
			Day 0	Day 14 pv	Day 30 pv	
25	Sham	F	<0.1	<0.1	<0.1	Y
8	Sham	F	<0.1	<0.1	<0.1	Y
27	Sham	M	<0.1	<0.1	<0.1	Y
28	Sham	F	<0.1	<0.1	<0.1	Y
29	Sham	M	<0.1	<0.1	<0.1	Y
30	Sham	M	<0.1	<0.1	<0.1	Y
13	Treatment	M	<0.1	<0.1 *	<0.1	N
14	Treatment	M	<0.1	<0.1	<0.1	N
10	Treatment	F	<0.1	<0.1	<0.1	N
20	Treatment	F	<0.1	<0.1	<0.1	N
26	Treatment	F	<0.1	<0.1	<0.1	N
16	Treatment	M	<0.1	<0.1	<0.1	Y
22	Treatment	F	<0.1	<0.1	<0.1	Y
3	Treatment	M	<0.1	<0.1	0.1	Y
5	Treatment	M	<0.1	0.1	0.1	Y
7	Treatment	M	<0.1	<0.1	0.2	Y
12	Treatment	F	<0.1	<0.1	0.3	Y
19	Treatment	F	<0.1	0.1	0.5	Y
23	Treatment	F	<0.1	<0.1	0.6	Y
18	Treatment	M	<0.1	0.1	0.9	Y
1	Treatment	M	<0.1	0.4	1.1	Y
6	Treatment	F	<0.1	3.3	1.3	Y
24	Treatment	F	<0.1	0.8	3.0	Y
11	Treatment	M	<0.1	1.2	10	Y

## Data Availability

The data presented in this study are available in this article and its Supplemental Material.

## Supplementary Materials

The following are available online at https://www.mdpi.com/article/10.3390/v13050734/s1, Figure S1: Geometric mean RVNA titers from mongooses offered ~1.8 mL 10^9.5^/mL TCID_50_ ONRAB oral rabies vaccine by bait (this study) verses DIOC [10] on day 14 pv, Figure S2: Geometric mean RVNA titers from mongooses offered ~1.8 mL 10^9.5^/mL TCID_50_ ONRAB oral rabies vaccine by bait (this study) verses DIOC [10] on day 30 pv.

## References

[1] Seetahal J.F.R., Vokaty A., Vigilato M.A.N., Carrington C.V.F., Pradel J., Louison B., Sauers A.V., Roopnarine R., Arrebato J.C.G., Millien M.F. (2018). Rabies in the Caribbean: A Situational Analysis and Historic Review. Trop. Med. Infect. Dis..

[2] Mueller T.F., Schroeder R., Wysocki P., Mettenleiter T.C., Freuling C.M. (2015). Spatio-temporal use of oral rabies vaccines in fox rabies elimination programmes in Europe. PLoS Negl. Trop. Dis..

[3] Slate D., Algeo T.P., Nelson K.M., Chipman R.B., Donovan D., Blanton J.D., Niezgoda M., Rupprecht C.E. (2009). Oral rabies vaccination in North America: Opportunities, complexities and challenges. PloS Neglect. Trop. Dis..

[4] Blanton J.D., Meadows A., Murphy S.M., Manangan J., Hanlon C.A., Faber M.-L., Dietzschold B., Rupprecht C.E. (2006). Vaccination of small Asian mongooses (*Herpestes javanicus*) against rabies. J. Wildl. Dis..

[5] Vos A., Kretzschmar A., Ortmann S., Lojkic I., Habla C., Müller T., Kasier C., Hundt B., Schuster P. (2013). Oral vaccination of captive small Indian mongoose (*Herpestes auropunctatus*) against rabies. J. Wildl. Dis..

[6] Rosatte R.C., Donovan D., Davies J.C., Allan M., Bachmann P., Stevenson B., Sobey K., Brown L., Silver A., Bennett K. (2009). Aerial distribution of ONRAB^®^ baits as a tactic to control rabies in raccoons and striped skunks in Ontario, Canada. J. Wildl. Dis..

[7] Slate D., Chipman R.B., Algeo T.P., Mills S.A., Nelson K.M., Croson C.K., Dubovi E.J., VerCauteren K., Renshaw R.W., Atwood T. (2014). Safety and immunogenicity of Ontario rabies vaccine bait (ONRAB) in the first US field trial in raccoons (*Procyon lotor*). J. Wildl. Dis..

[8] Gilbert A.T., Johnson S.R., Nelson K.M., Chipman R.B., VerCauteren K.C., Algeo T.P., Rupprecht C.E., Slate D. (2018). Field trials of Ontario rabies vaccine bait in the northeastern USA, 2012–14. J. Wildl. Dis..

[9] Pedersen K., Gilbert A.T., Nelson K.M., Morgan D.P., Davis A.J., VerCauteren K.C., Slate D., Chipman R.B. (2019). Raccoon (*Procyon lotor*) response to Ontario Rabies Vaccine Baits (ONRAB) in St. Lawrence County, New York, USA. J. Wildl. Dis..

[10] Berentsen A.R., Ellis C.K., Johnson S.R., Leinbach I.L., Sugihara R.T., Gilbert A.T. (2020). Immunogenicity of Ontario rabies vaccine for small Indian mongooses (*Herpestes auropunctatus*). J. Wildl. Dis..

[11] Berentsen A.R., Johnson S.R., VerCauteren K.C. (2014). Bait matrix flavor preference by mongoose (*Herpestes auropunctatus*) in Puerto Rico: Implications for oral rabies vaccination. Caribb. J. Sci..

[12] Yager M.L., Moore S.M., Rupprecht C., Hagarajan T. (2015). The rapid fluorescent focus inhibition test. Current Laboratory Techniques in Rabies Diagnosis, Research and Prevention.

[13] R Core Team (2019). R: A Language and Environment for Statistical Computing.

[14] Blancou J., Schneider L.G., Wandeler A., Pastoret P.P. (1985). Vaccination du renard roux (*Vulpes vulpes*) contre la rage par voie orale. Bilan d’essais en station expérimentale. Rev. Ecol. Terre Vie.

[15] Moore S.M., Gilbert A., Vos A., Freuling C.M., Ellis C., Kliemt J., Müller T. (2017). Rabies virus antibodies from oral vaccination as a correlate of protection against lethal infection in wildlife. Trop. Med. Infect. Dis..

[16] Te Kamp V., Freuling C.M., Vos A., Schuster P., Kaiser C., Ortmann S., Kretzschmar A., Nemitz S., Eggerbauer E., Ulrich R. (2020). Responsiveness of various reservoir species to oral rabies vaccination correlates with differences in vaccine uptake of mucosa associated lymphoid tissues. Sci. Rep..

[17] Berentsen A.R., Sugihara R.T., Payne C.G., Leinbach I., Volker S.F., Vos A., Ortmann S., Gilbert A.T. (2019). Iophenoxic acid as a biological marker for oral rabies vaccination in the small Indian mongoose (*Herpestes auropunctatus*). J. Vis. Exp..

[18] Berentsen A.R., Chipman R.B., Nelson K.M., Gruver K.S., Boyd F., Volker S.F., Davis A.J., Vos A., Ortmann S., Gilbert A.T. (2020). Placebo Oral Rabies Vaccine Bait Uptake by Small Indian Mongooses (*Herpestes auropunctatus*) in Southwestern Puerto Rico. J. Wild. Dis..

