# Supplementary material for: Oral Rabies Vaccination of Small Indian Mongooses (Urva auropunctata) with ONRAB via Ultralite Baits
Source: Viruses. 2021 Apr 23;13(5):734. doi: 10.3390/v13050734 (PMC8144982; doi:10.3390/v13050734)
Supplement: Supplementary file 1 [file viruses-13-00734-s001.zip › viruses-1165015-supplementary.pdf]

viruses MDPI

Supplementary Material

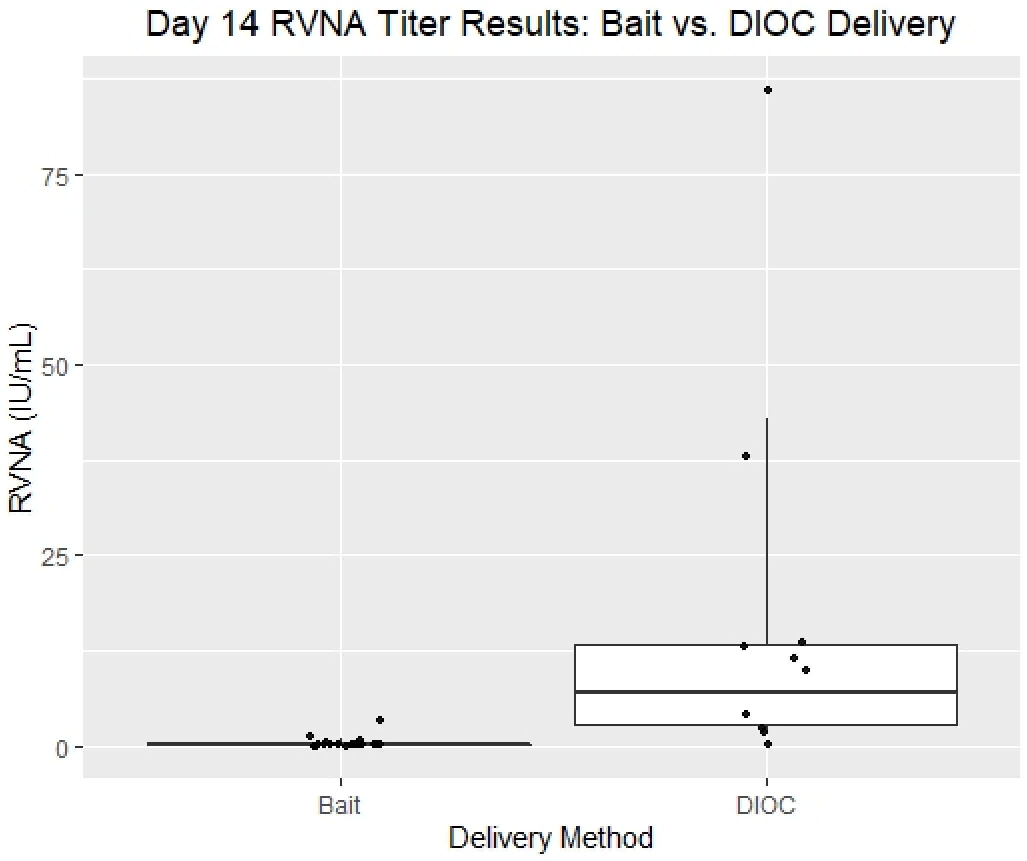

Figure S1: Geometric mean RVNA titers from mongooses offered ~1.8 mL $10^{9.5}$/mL $TCID_{50}$ ONRAB oral rabies vaccine by bait (this study) verses DIOC [10] on day 14 pv.

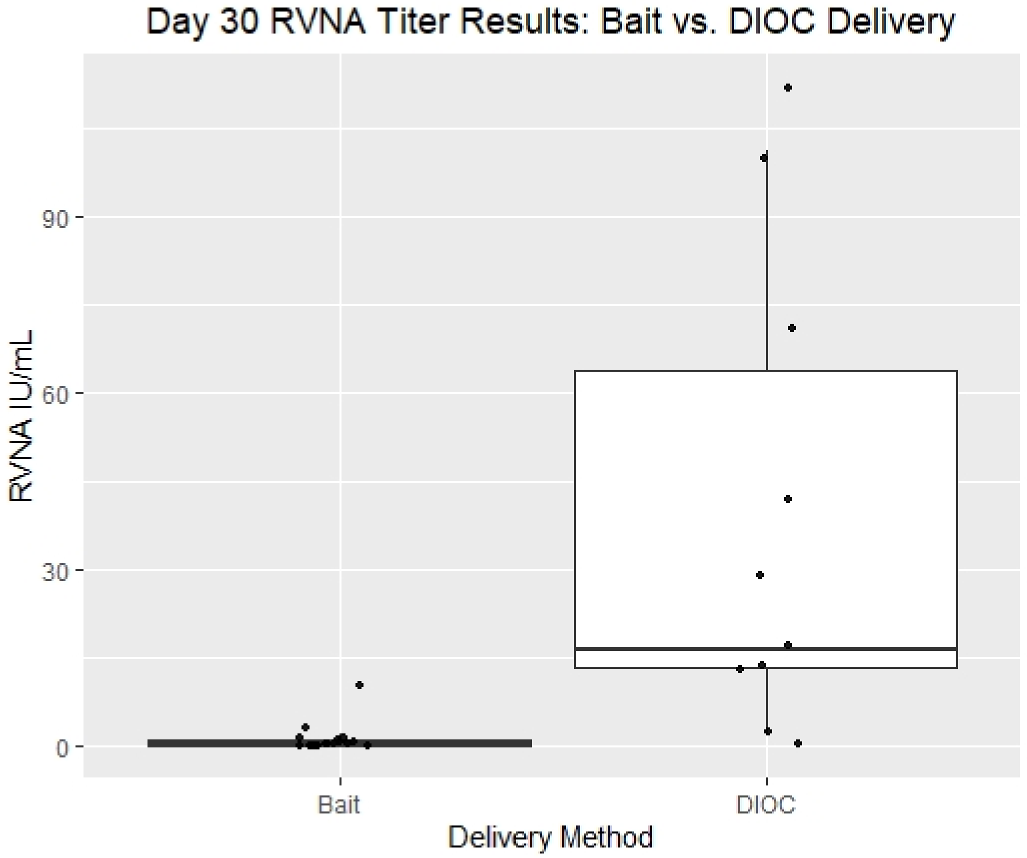

Figure S2: Geometric mean RVNA titers from mongooses offered ~1.8 mL $10^{9.5}$/mL $TCID_{50}$ ONRAB oral rabies vaccine by bait (this study) verses DIOC [10] on day 30 pv.
